# Influence of substructure material on the scanning accuracy and scannability of implant-supported full arch bar substructures

**DOI:** 10.1038/s41598-025-28419-2

**Published:** 2025-12-03

**Authors:** Nourhan S. Emam, Nayrouz Adel Metwally, Mohamed Moataz Khamis

**Affiliations:** 1 Lecturer of Prosthodontics, Faculty of Dentistry, Zagazig University, AlSharqia, Egypt; 2 Department of Prosthodontics, Faculty of Dentistry, Alexandria University, Alexandria, Egypt; 3 Ph.D Researcher at the Department of Prosthodontics, Faculty of Dentistry, Alexandria University, Alexandria, Egypt

**Keywords:** Scannability, Digitization, Accuracy, PEEK bar, Titanium bar, Implant-supported prosthodontics, Trueness and precision, Health care, Medical research

## Abstract

Bar substructures may be utilized for fixed full-arch implant-supported restorations, where suprastructures are cemented as prosthetic shells. Certain clinical situations may necessitate intraoral scanning of the bar substructures. However, the scannability and the accuracy of the intraoral scans remain unclear. This study aimed to compare and assess the scannability and the scan accuracy of two different bar materials used for implant-supported prostheses. Two maxillary implant-supported substructures (bars) were milled from 2 different materials. Group I from titanium and Group II from poly-ether-ether-ketone (PEEK). The substructures were digitized by using a desktop scanner (Medit MD-1D0410, Medit Corp). The STL file produced was considered the reference. Each substructure was scanned intraorally (*n* = 10) by using an intraoral scanner (IOS) (Medit i700; Medit Corp). The non-captured surface area in a preset time limit of 15 s (scannability) was assessed in (mm^2^). To evaluate the scanning accuracy, all STL files were imported into a surface-matching software program (Medit Design v3.0.6 Build 286; Medit Corp), where overall RMS (root mean square) deviations in (µm) were calculated. The Shapiro–Wilk test of normality was used. A comparison between the study groups was done by using an independent samples t-test. Significance was set at *P* < .05. The titanium substructure demonstrated better trueness and precision concerning deviation analysis (202.40 ± 27.57 and 197.50 ± 24.69, respectively) than the PEEK substructure (262.20 ± 30.87 and 244.1 ± 9.18, respectively) (*P* < .001). Regarding scannability, significantly more surface area was captured when scanning the PEEK substructure (985.42 ± 7.22) than titanium (951.15 ± 12.16) (*P* < .001). PEEK was more scannable. The titanium bar demonstrated significantly higher accuracy than the PEEK bar, which was more scannable than the titanium bar.

This trial, number NCT06423482, is registered at Clinical.gov. Its start date was May 14, 2024.

**Supplementary Information:**

The online version contains supplementary material available at 10.1038/s41598-025-28419-2.

## Introduction

Several factors can affect the accuracy of intraoral scanning, including the type of scanner, device calibration, scanning pattern, ambient illumination, humidity, and particular scanning conditions. The optical qualities of the digitized material surface also influence the scan accuracy^1–15^.

According to the International Organization for Standardization (ISO) 5725 standard, trueness and precision determine IOS accuracy^6,17,17^. While precision is the degree of agreement between independently measured values in a simulated environment representing the repeatability of measurements, trueness is the agreement between a test result and a known reference value^6,18^.

Scannability is defined as the ability of the surface of a material to be entirely scanned by a digital scanner in a specific time frame^19^. It is assessed by the calculated scanned/captured surface area for a specific surface/material in a predetermined time frame, measured in mm^2^/Sects^19,20^. It is affected by the material reflectiveness, translucency, surface texture, scanner type, surface treatment, wettability, and use of scanning aid materials^21^. Scannable materials are scanned without the need for a surface anti-reflective powder^22,24,24^.

Full-arch fixed implant-supported restorations can be constructed using various design options and materials. A feasible prosthetic option is a CAD-CAM milled suprastructure passively luted to a bar substructure. During the digital workflow of prosthesis construction, the CAD software program designs both supra and substructures. The software then reverse engineers the bar by extracting it from the original design^25^.

Studies have reported that scanning the bar after production, before suprastructure fabrication, improves passive fit and adaptation over the bar that may have undergone dimensional changes during machining^25^. Desktop scanners in the lab can be used for that purpose^26,28,28^.

In the clinical situation, the metal substructure is usually delivered with a PMMA suprastructure during the try-in phase^25^. Adjustments can be made to the polished surface of the metal substructure if necessary, and the PMMA suprastructures can be modified according to aesthetic and phonetic requirements, as well as occlusal relationships. The assembly can also be used temporarily by the patient until the final prosthesis is fabricated. Two intraoral scans are required in a fully digital workflow: One for the metal substructure and the other for the PMMA suprastructure that is placed on top of it^29,30^. Bar substructures can be made of different materials, commonly titanium and PEEK. While PEEK offers a lightweight, metal-free alternative with advantageous optical properties that can enhance scannability, titanium has traditionally been utilized for its strength and clinical reliability. Comparing their scanning accuracy and scannability is therefore relevant for optimizing digital full-arch implant workflows.

The scannability and accuracy of titanium and PEEK were previously tested^19^. Titanium exhibited higher trueness, while PEEK was more precise and more scannable than titanium. Previous studies have also reported that intraoral conditions moderately affected the precision and trueness of intraoral scanning^31^.

The scanning accuracy and scannability of the prosthesis substructure are essential to ensure a passively fitting suprastructure^32^. The present study compared the intraoral scanning accuracy and scannability of substructures fabricated from titanium versus PEEK^19^. The null hypothesis was that no significant difference would be found in the accuracy or the scannability between the 2 studied groups.

## Methods

**Fig. 1:**
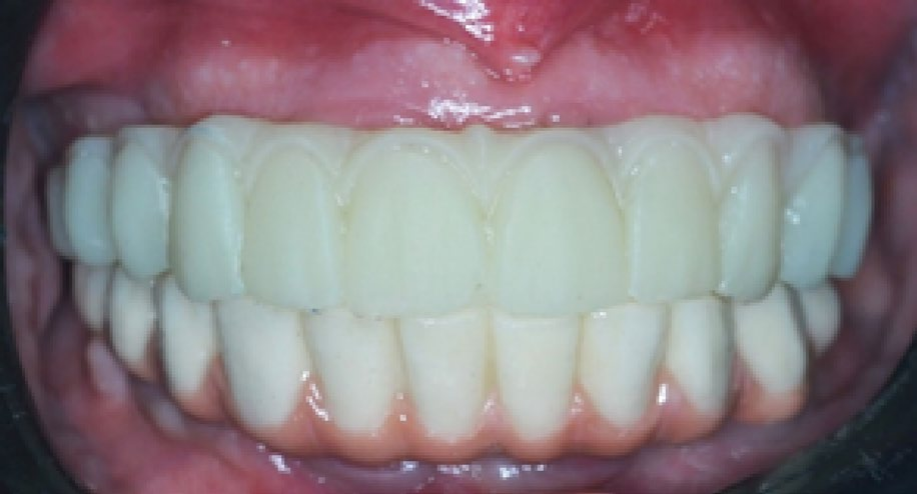
A fully anatomic try-in for esthetics and occlusion verification.

**Fig. 2:**
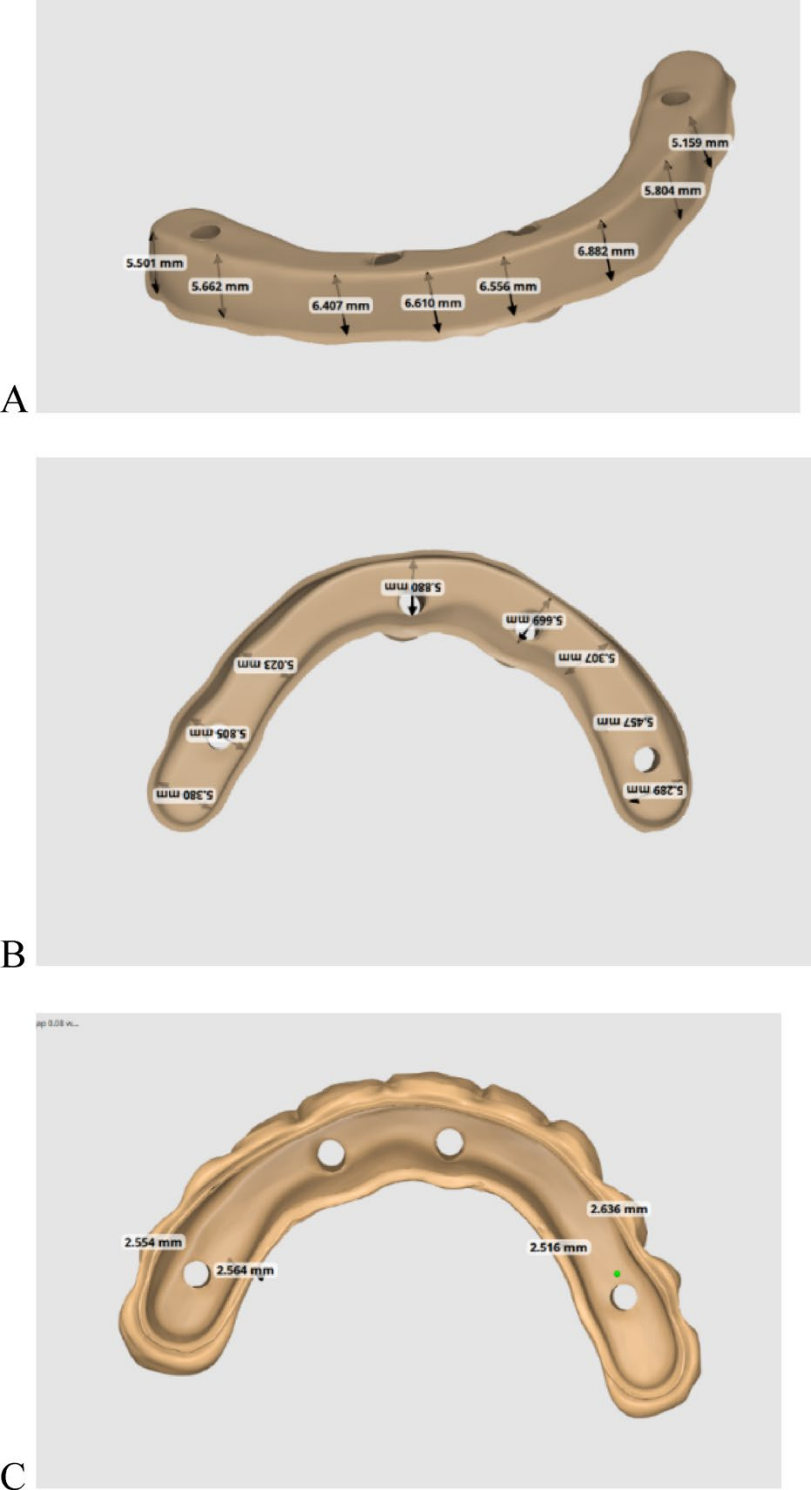
Supra and substructure dimensions. (**A**) Occluso-cervical height. (**B**) Bucco-palatal width. (**C**) 2.5 mm thick suprastructure dimensions.

A maxillary edentulous patient having 4 osseointegrated implants (Vitronex Elite, Vitronex Implant system, Italy) was selected for the study. Multiunit abutments (MUA, Vitronex Elite) were selected, tightened, and torqued to the implants. Scan bodies (MUA Scan body, Vitronex Elite) were tightened to the abutments following the manufacturer’s guidelines. A chairside digital impression of the maxillary arch was made using an intraoral scanner (Medit i700; Medit Corp). A verification jig was made to verify the accuracy of the digital impression. A physical model of the virtual impression was 3D printed (Phrozen water-washable dental cast resin; Phrozen Tech Co, Ltd) by using resin (Phrozen Sonic Mini 4 K; Phrozen Tech Co, Ltd). A full arch screw-retained restoration was designed for the patient by using a CAD software program (Blender for Dental v3.6; Blender Foundation, B4D iBar™ module)^33,34^. A fully anatomic try-in was 3D printed (MAMMOTH 3D printer 6.6, V-Ceram Shop) from temporary resin material (Pro shape, Temp Resin, Turkey) as shown in Fig. 1. It was tried intraorally to verify esthetics and occlusion. The CAD design was then split into a bar substructure and suprastructure. The bar substructure had buccal and lingual finish lines with a width of 1 mm, as shown in Fig. 2.

**Fig. 3:**
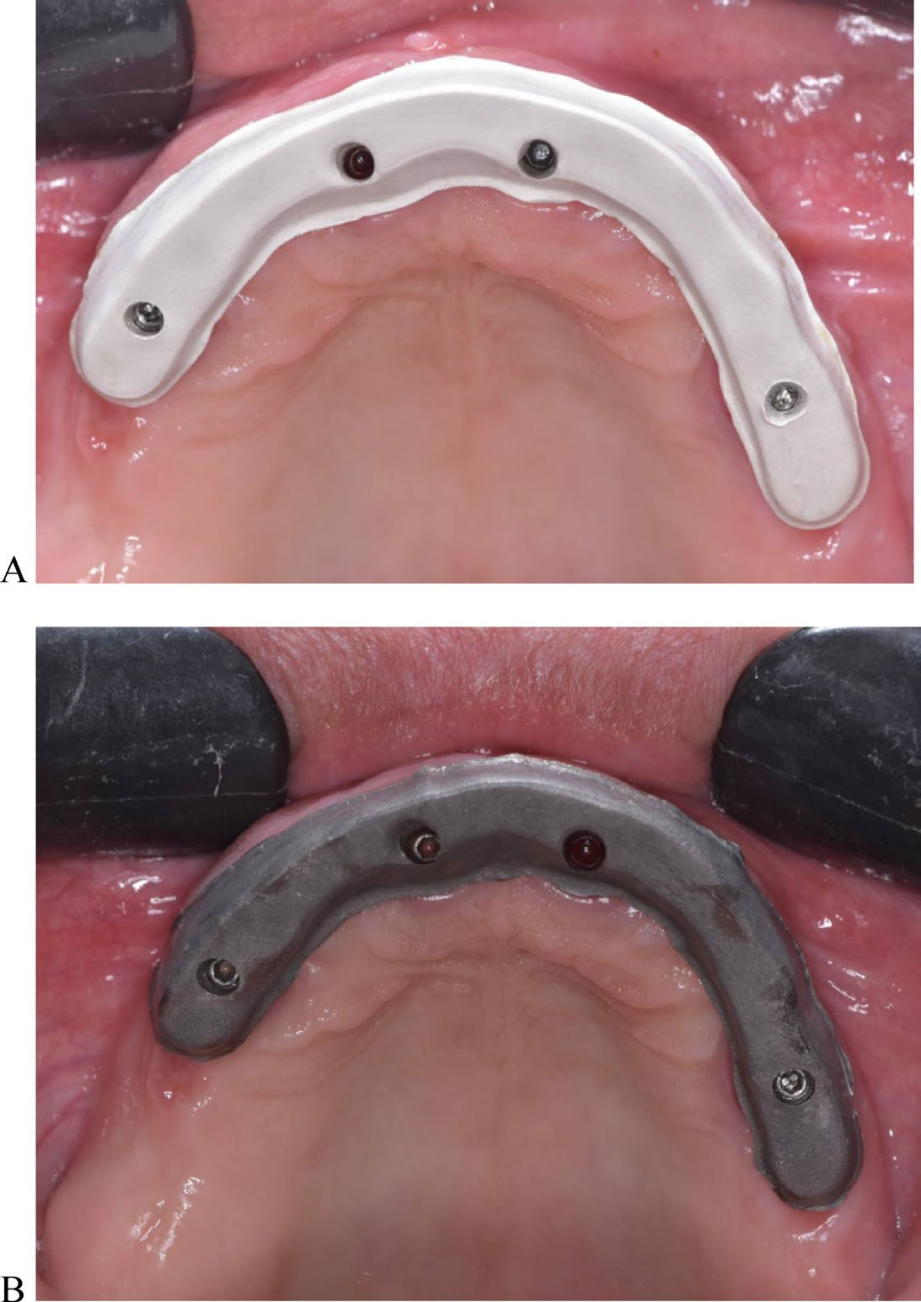
Milled bars intraoral try-in. (**A**) airborne particle abraded PEEK bar. (**B**) airborne particle abraded Titanium bar try-in.

Two groups were formed based on the substructure material: PEEK (breCAM, BioHPP disk, Bredent GmbH & Co. KG, Senden, Germany) and titanium (dentatec, GmbH, Germany). The inter-arch space and the arch width allowed for a bar height of 5.1–6.6 mm occluso-cervically and a width of 5–6 mm buccolingually^35,36^. The STL file of the virtual substructure was exported for milling titanium and PEEK bars by using a milling machine (Roland DWX-52D Plus, Roland DGA Corporation). Bars were evaluated for passivity on the printed model, after which they were tried intraorally, as shown in Fig. 3. The one-screw test was used to ensure their passivity^31^.

Airborne-particle abrasion was used to roughen both bars using 50 μm Al_2_O_3_ (Aluminum oxide, Eisenbacher Dentalwaren; ED GmbH). Abrasion was completed at 0.2 MPa pressure from a 10 mm distance for 1 min^31,34,37,38^.

### Accuracy assessment

**Fig. 4:**
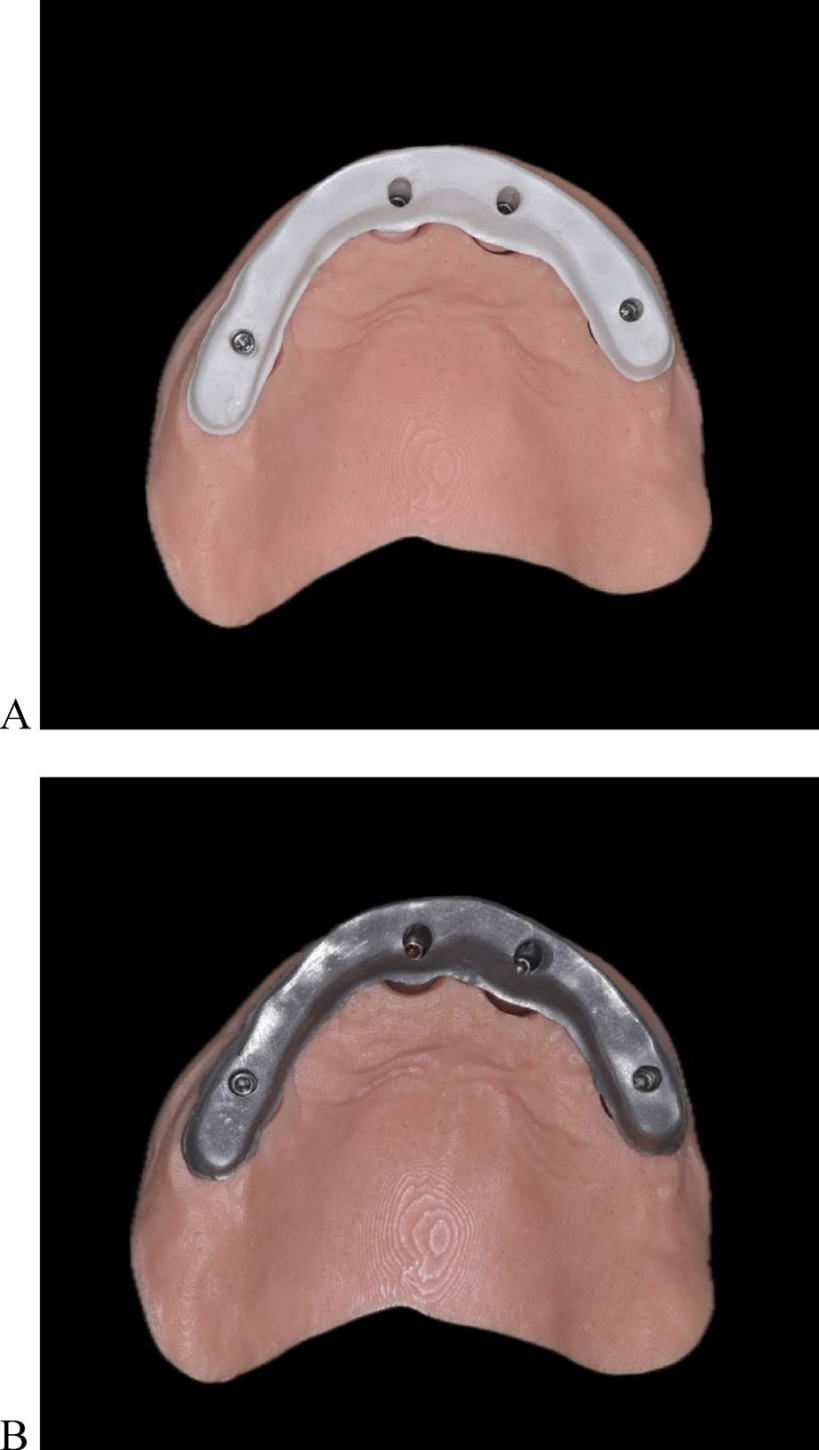
Bar substructures try-in on a 3D-printed model. (**A**) PEEK bar, and (**B**) titanium bar.

Each bar substructure was scanned on the 3D-printed model by using a desktop scanner (Medit MD-1D0410, Medit Corp) to create a reference file STL_R_ to be used as a control for each group, as shown in Fig. 4. Each bar was scanned 10 times intraorally by using an IOS (Medit i700; Medit Corp) according to the calculated sample size. Both groups followed the identical scan pattern, which began at the palatal surface, proceeded to the occlusal side, and terminated on the buccal side (P-O-B)^39^. A single experienced operator performed the scanning procedure to standardize the scanning speed, distance, and operator experience. Ambient light was adjusted to 1000 lx^40^.

The STL_R_ file acquired from scanning the bar using the desktop scanner was used as a reference scan to evaluate trueness. Intraoral scans were aligned over the reference scan by using the best fit feature. The bar was selected as the reference. It was used to match its corresponding STL_I_, which was assigned as the target with the automatic alignment algorithm. To evaluate precision, deviations for all unique STL file pairings were calculated without repetition (45 comparisons). Each group included ten intraoral scans, resulting in 45 possible combinations. Then, 45 RMS values were obtained using the best-fit algorithm and three-dimensional comparison for each pair. The average of these 45 RMS values indicates the precision of a single group. Therefore, the precision of each group was determined^19,41^.

**Fig. 5:**
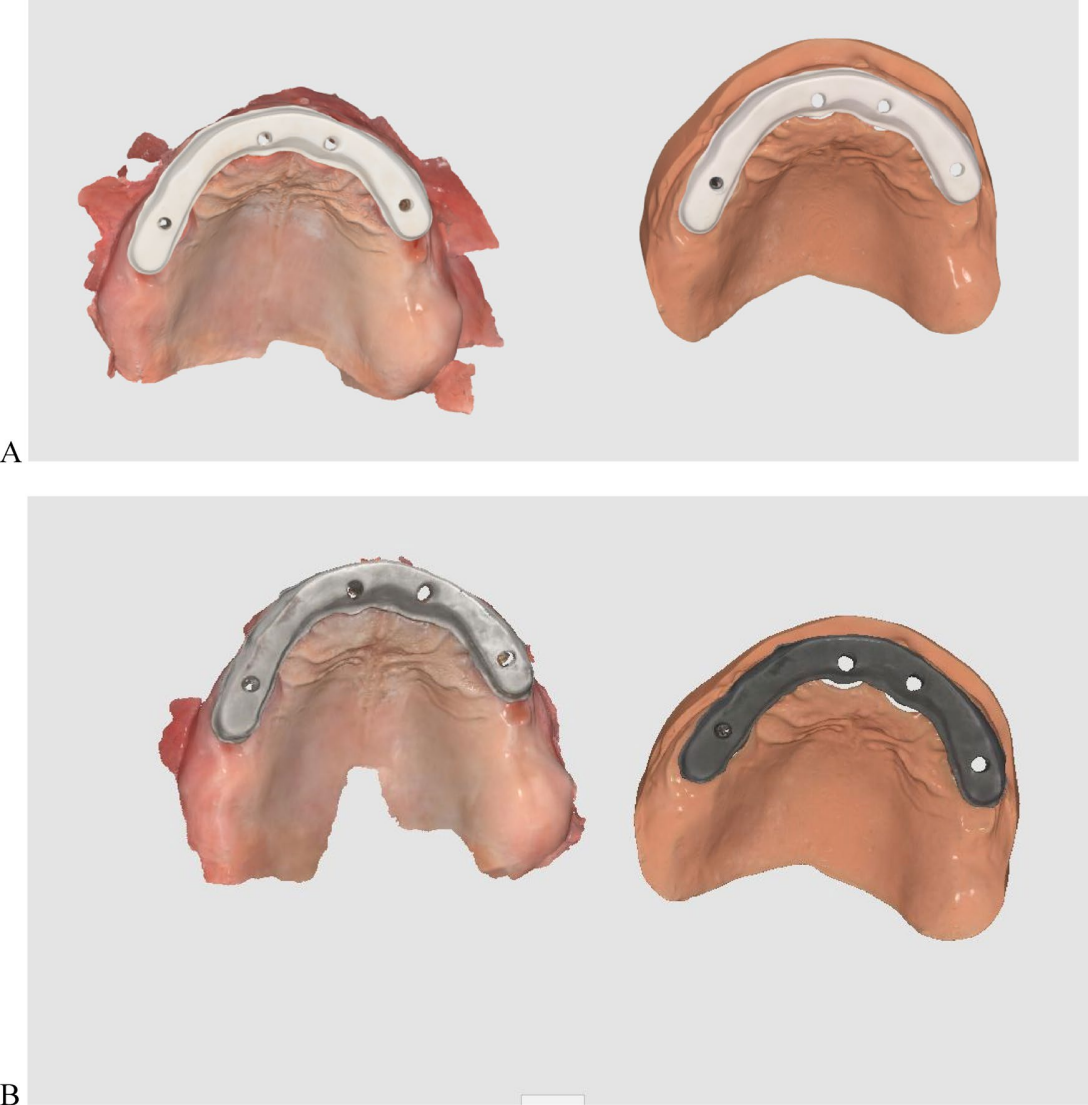
Scanned bars (reference and test scans) before alignment for trueness assessment.

**Fig. 6:**
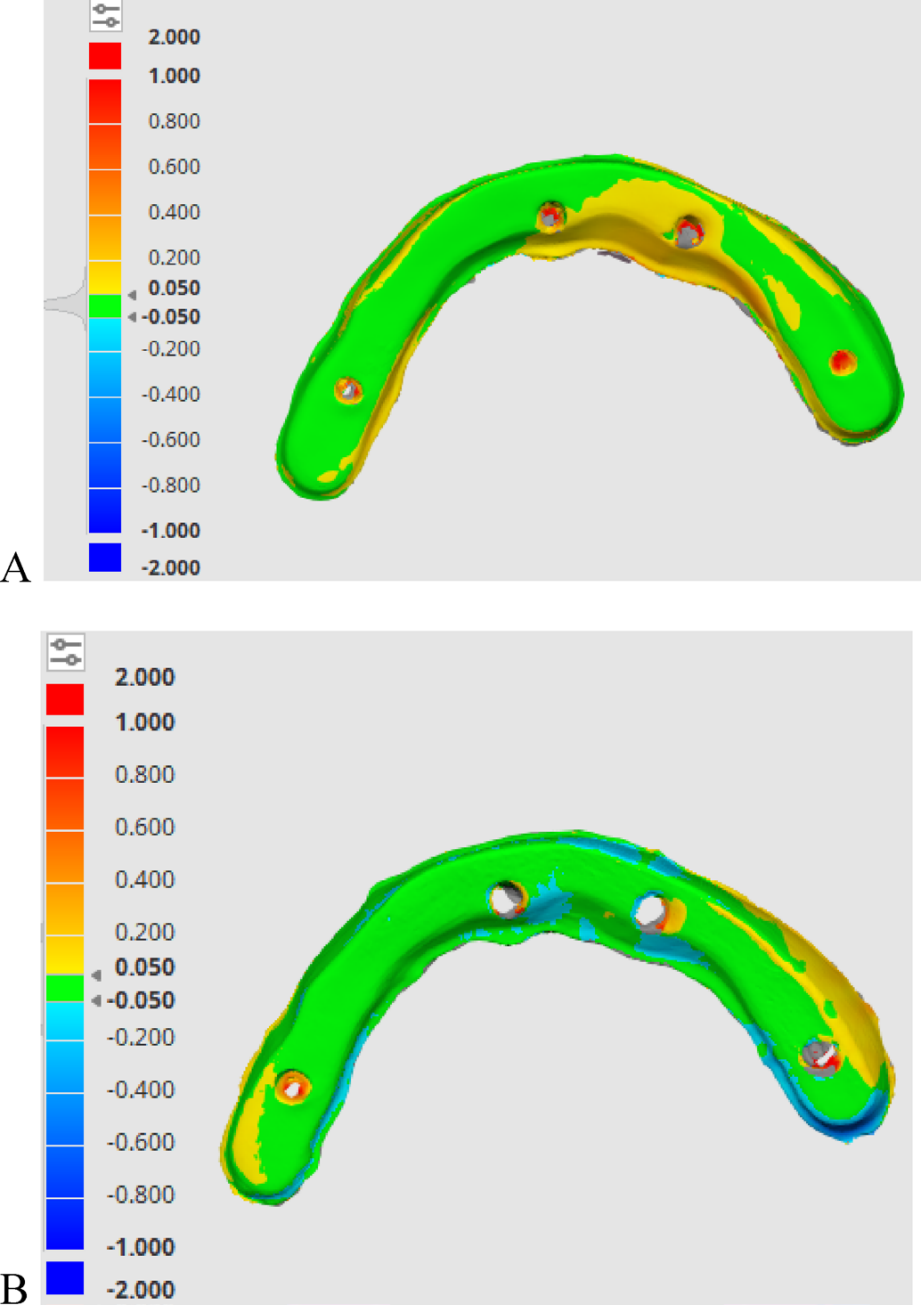
Trueness is demonstrated by a color-coded map determining the deviation between the two meshes (test and reference scans). (**A**) PEEK bar, and (**B**) titanium bar.

**Fig. 7:**
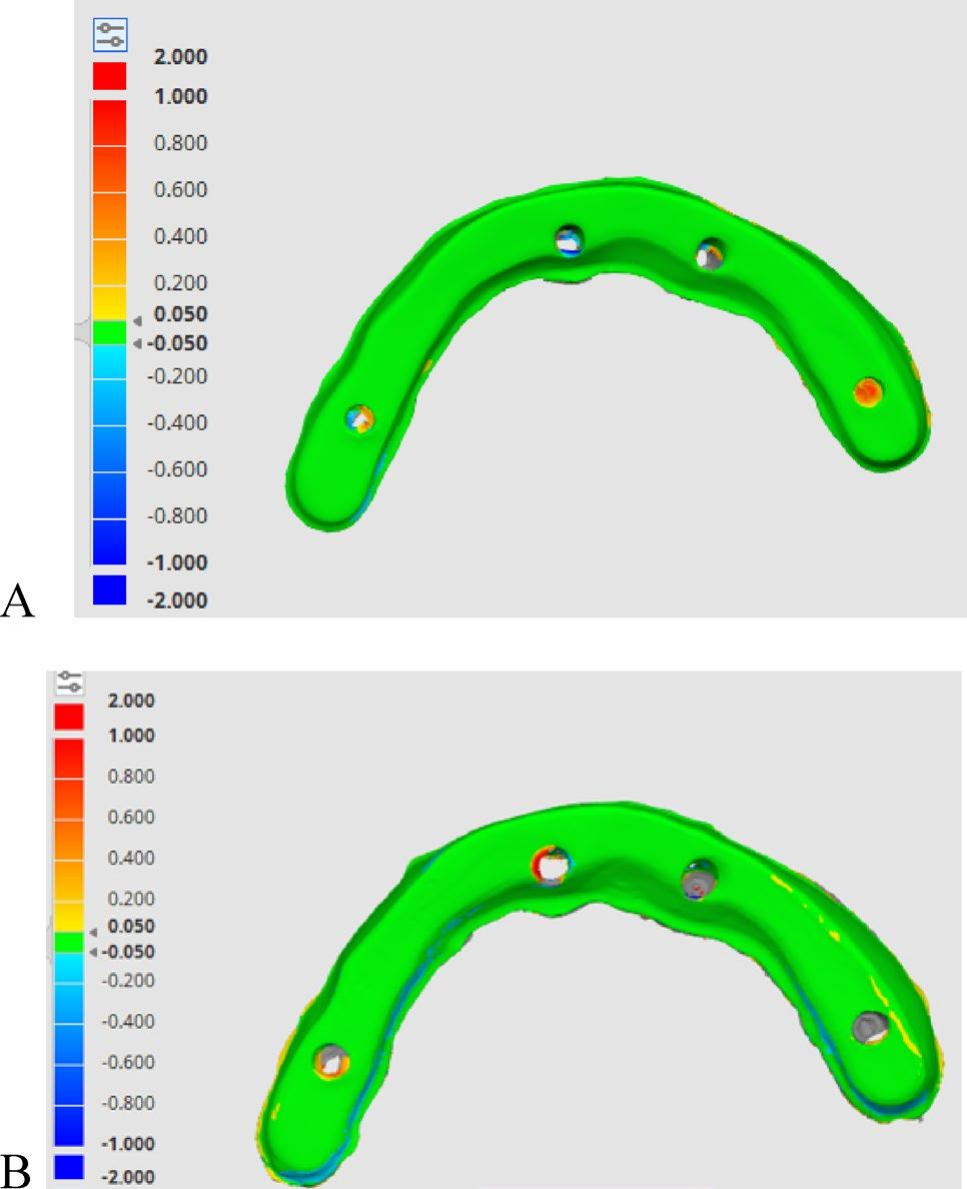
A demonstrative color-coded map that illustrates precision within the same group. (**A**) PEEK bar, and (**B**) Titanium bar.

Accuracy was evaluated using a non-metrology grade software program (Medit Link Compare Tool, Medit Design v3.0.6, Build 286; Medit Corp), as shown in Fig. 5. For the quantitative assessment of 3D discrepancies between the target data STL_I_ and the reference data STL_R_ for both titanium and PEEK bars, the deviation display mode of the software was used to generate color-difference maps. From these maps, the overall root mean square (RMS) values were calculated and used for the statistical analysis of trueness and precision^19,42,44–48,48^, as shown in Figs. 6 and 7. The deviation threshold for green (representing minimal discrepancies) was set between − 50 μm and + 50 μm as shown in Figs. 6 and 7. The minimum and maximum deviation ranges (green color) were set at −50 μm and + 50 μm, respectively^42,43^.

### Scannability assessment

According to the Emam et al. technique, scannability was evaluated^19^. Titanium and PEEK bars were scanned individually to assess scannability. The minimum time in seconds necessary to thoroughly scan each bar was first calculated by intraorally scanning every bar. The PEEK bar was thoroughly scanned in 16 s, whereas the titanium bar required 20 s. The STL files of the thoroughly scanned bars were saved as a reference to evaluate scannability later on. A (T-1) approach was followed (reference) so that (T) was the time necessary to scan the most scannable bar material completely and defect-free (as PEEK was fully scanned in 16 s, it was used as the reference for calculating T). One second was subtracted from the estimated time T (T-1).

**Fig. 8:**
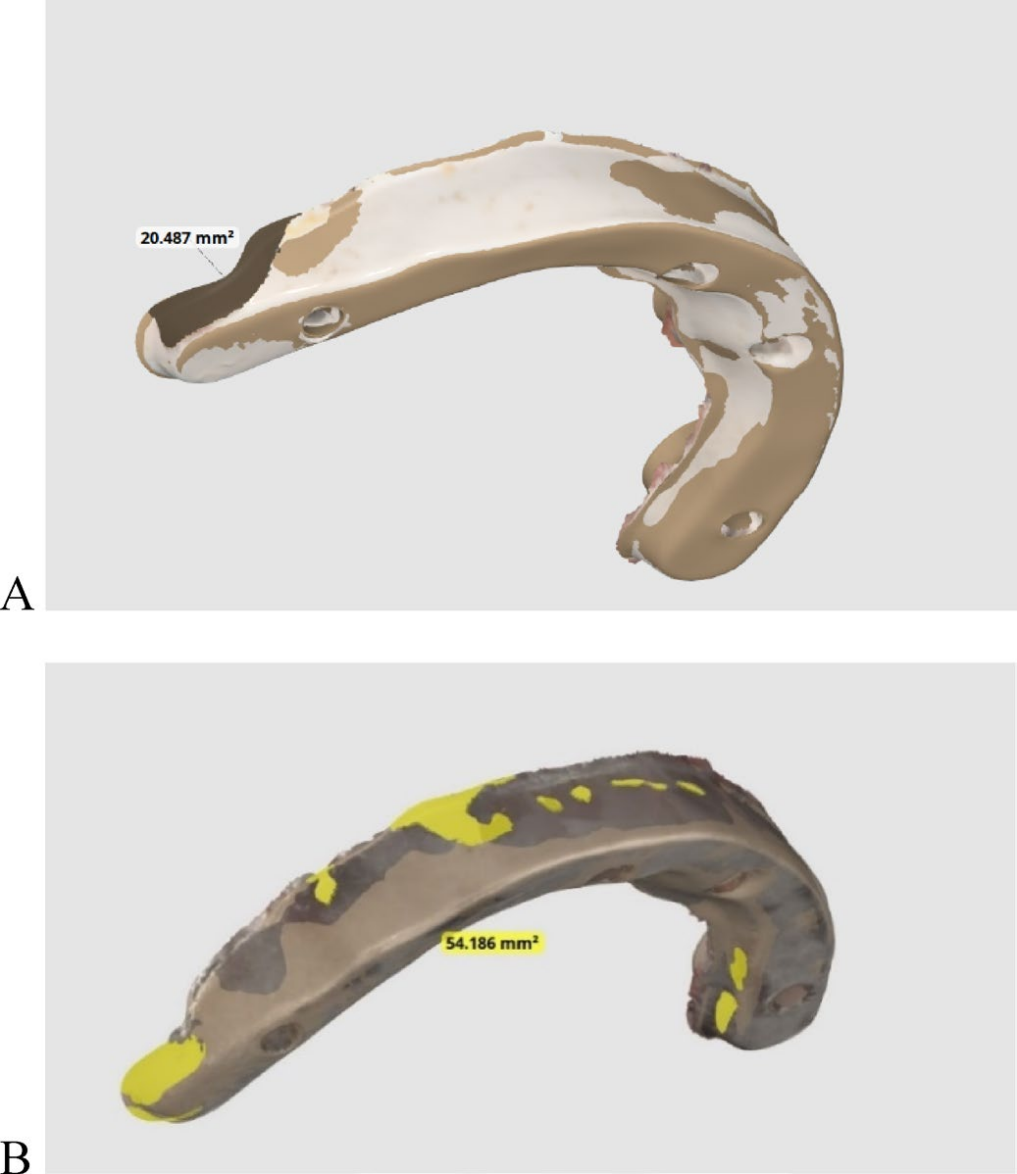
Scannability assessment by calculating the non-captured area in mm^2^ in a predetermined time frame of 15 s for (**A**) PEEK Bar (missing areas in black). (**B**) titanium bar (missing areas in yellow).

A scanning time limit of 15 s (T-1) was selected to evaluate each material’s sensitivity to rapid, complete, and defect-free scanning (scannability). The 2 substructures were scanned, each 10 times, every time in 15 s. With a total of 20 scans for both bars, the missing surface area for each substructure was determined after matching the STL file for every group with the missing fragments with the virtual reference STL file previously obtained for every group., The total surface area was 1000.739 mm^2,^ as shown in Fig. 8. The Automatic alignment mode was chosen for superimposition (The Medit link compare tool, Medit Design v3.0.6, Build 286; Medit Corp), with the virtual design as the reference file and the scan in a predetermined time frame as the target file to measure the area of missing polygons (Fig. 8)^19^.

The reference file (virtual design) was used in STL format, while the tested file (bar) was used in PLY (Polygon File Format) to view the missing polygons easily after superimposition. The area of non-captured areas was calculated and tabulated^19^.

Sample size estimation was performed to allow statistical comparison with a study power of 80% and a significance level (α) of 0.05, based on the effect size (2.154) reported by Emam et al.^19^ a minimum of 10 digital scans per group was required. Accordingly, 20 scans were obtained (10 scans × 2 groups). All outcomes showed statistically significant differences, and post-hoc power analysis confirmed that the achieved power exceeded 95% for all comparisons, indicating that the sample size was sufficient to support the study findings.

Data were collected and statistically analysed with a statistical software program (IBM SPSS Statistics for Windows, v23.0; IBM Corp) 5. Normality was checked for all variables by using the Shapiro–Wilk test. All variables showed normal distribution, so means and standard deviation (SD) were calculated, and parametric tests were used. Comparisons between the 2 study groups were performed using an independent samples t-test to calculate adjusted means, standard error (SE), and 95% CIs. Significance was set at (*P* <.05). This trial is registered at Clinical.gov, NCT06423482, started May 14, 2024.

## Results

**Fig. 9:**
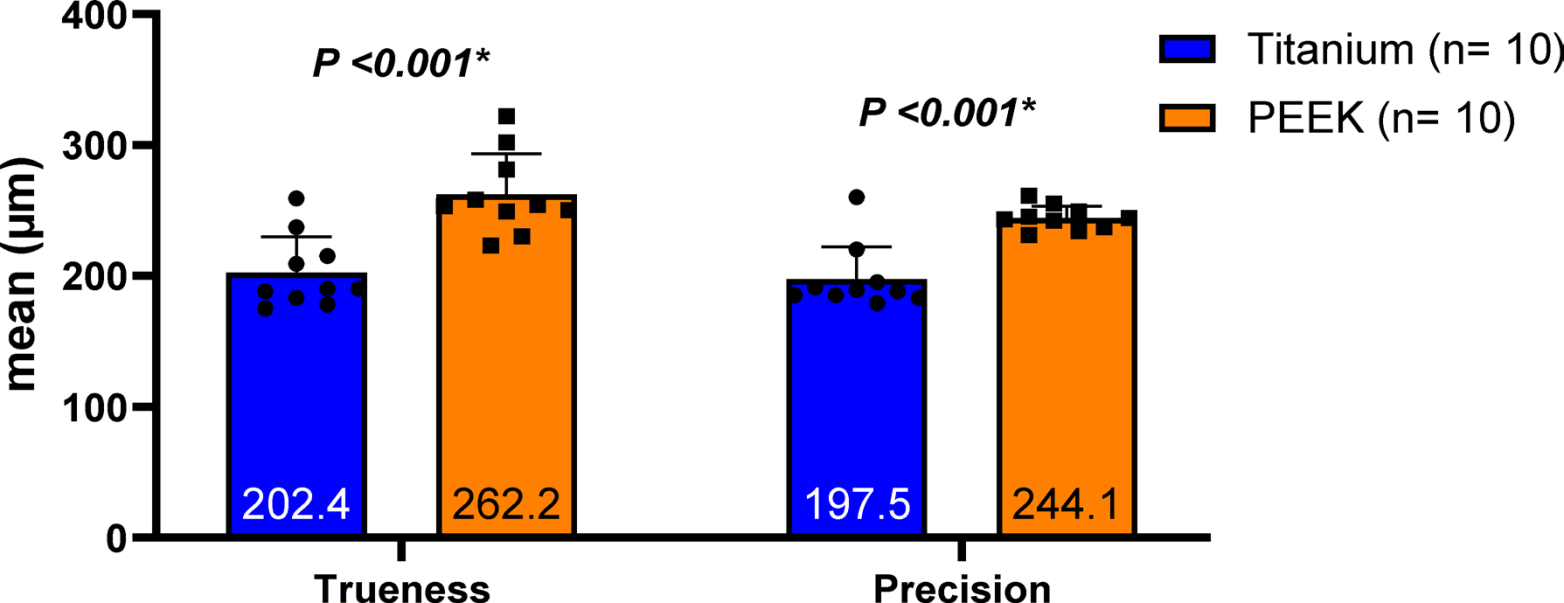
Comparative analysis of the PEEK and titanium groups’ overall RMS in µm for precision and trueness.

The overall RMS in µm was used to represent precision and trueness. Deviation in trueness in the studied groups is presented in Table 1. The mean and standard deviation of trueness of the titanium bar (202.40 ± 27.57) were less than that of the PEEK bar (262.20 ± 30.87) (*P* <.001), with a significant difference. Deviation in precision in the different studied groups is presented in Table 1. The titanium bar was significantly more precisely scanned (197.50 ± 24.69) compared to the PEEK bar (244.1 ± 9.18) (*P* <.001), as shown in Fig. 9.

**Table 1:** Comparison of trueness and precision (µm) between the two study groups.

		Titanium	PEEK	Mean difference (95% CI)	T-test *P* value
Trueness	Mean (SD)	202.40 (27.57)	262.20 (30.87)	−59.80 (−87.30, −32.30)	T = 4.57 *P* <.001*
	Median (IQR)	190.00 (38.75)	253.50 (42.00)		
	95% CI	182.68, 222.12	240.11, 284.29		
Precision	Mean (SD)	197.50 (24.69)	244.10 (9.18)	−46.60 (−64.85, −28.35)	T = 5.59 *P* <.001*
	Median (IQR)	188.50 (16.75)	243.50 (14.25)		
	95% CI	179.83, 215.17	237.53, 250.67)		

SD: Standard Deviation, IQR: Interquartile range, CI: Confidence Interval.

Independent samples t-test was used.

*Statistically significant at *P* <.05.

**Fig. 10:**
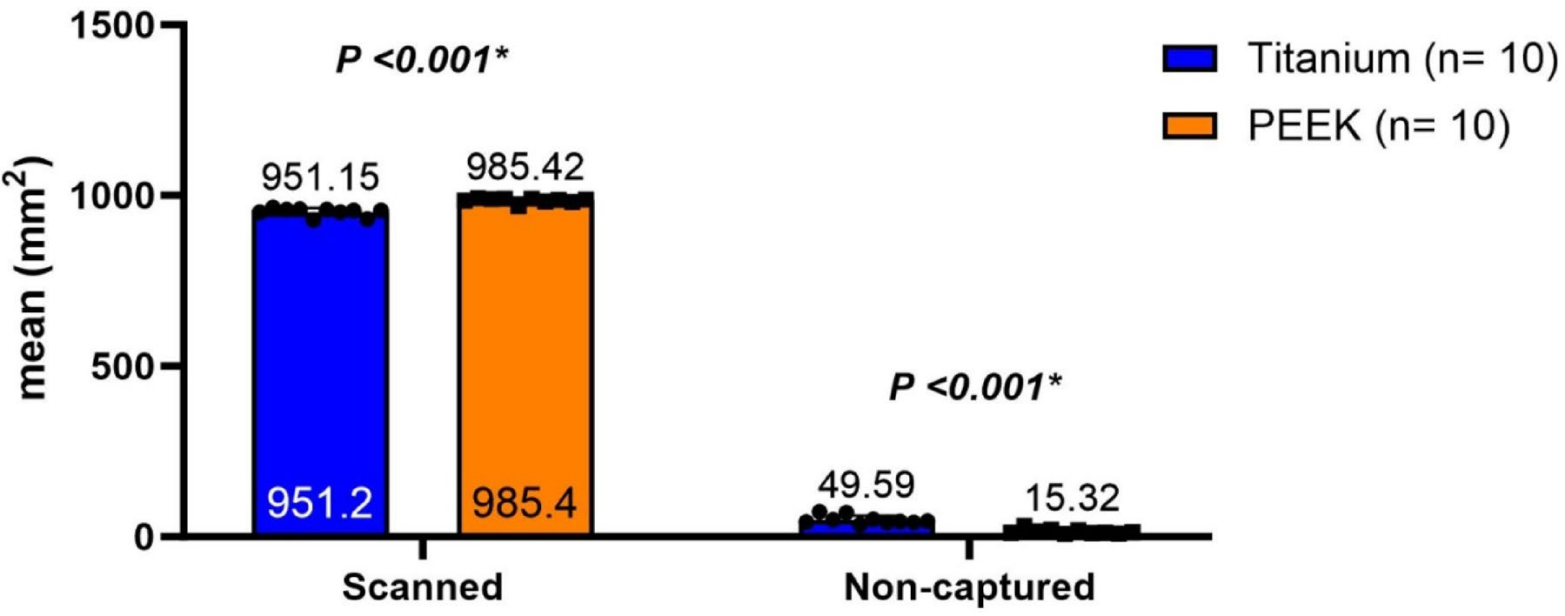
Comparative analysis of scannability between PEEK and titanium via mm^2^ of the scanned Vs. Non-captured surface area in a predetermined time frame of 15 s.

A scannability comparison between PEEK and titanium through the non-captured surface area (in mm^2^) in a predetermined time frame is presented in Table 2. The area of the scanned surface in a predetermined time frame of 15 s was significantly more in the PEEK group (985.42 ± 7.22) than in the titanium group (951.15 ± 12.16) (*P* <.001); PEEK was more scannable than titanium in a limited time frame, as shown in Fig. 10.

**Table 2:** Comparison of scannability between the two study groups.

			Titanium	PEEK	Mean difference (95% CI)	t-test *P* value
Non-captured	Surface area	Mean (SD)	49.59 (12.16)	15.32 (7.22)	34.26 (24.87, 43.66)	t = 7.66 *P* <.001*
		Median (IQR)	45.11 (13.01)	12.75 (9.46)		
		95% CI	40.89, 58.28	10.16, 20.49		
Scanned	Surface area	Mean (SD)	951.15 (12.16)	985.42 (7.22)	−34.26 (−43.66, −24.87)	t = 7.66 *P* <.001*
		Median (IQR)	955.63 (13.01)	987.99 (9.46)		
		95% CI	942.46, 959.85	980.25, 990.58		

SD: Standard Deviation, IQR: Interquartile range, CI: Confidence Interval.

Independent samples t-test was used.

*Statistically significant at *P* <.05.

## Discussion

This research indicates that the type of prosthodontic material significantly impacts scanning accuracy, as measured by trueness and precision, and scannability of the evaluated prosthodontic materials. Consequently, the null hypothesis was rejected.

In case of full-arch implant-supported bars, we may use intraoral scanners to scan the bar substructure^49^. However, prior research has demonstrated that, in contrast to in vitro scanning, the accuracy of dental arches in vivo was significantly reduced, affected by clinical conditions^31,50^.

The present clinical research investigated the influence of scanning various implant-supported prosthodontic substructure materials on their digitization accuracy and scannability.

The current findings indicate that PEEK displays enhanced scannability, whereas titanium achieves superior scan accuracy. These results emphasize the distinction between ease and speed of scanning (scannability) and the dimensional accuracy of the resulting digital model. Clinically, although PEEK’s optical properties may facilitate faster and smoother scanning, titanium’s higher accuracy remains critical for achieving precise prosthesis fit. Therefore, material selection should strike a balance between scanning efficiency and the accuracy required for definitive fabrication in full-arch implant workflows.

To ensure consistency and avoid potential confounding variables related to scanner type, all tests in this study were standardized using a single scanner.

If the bar was modified chairside, intraoral scanning captures this updated version^32^, reducing clinical steps and improving patient comfort. This approach also follows the manufacturer’s recommended protocol, which includes scanning the soft tissue first and then the bar in situ to optimize the gingival contours of the suprastructures^51^.

Several approaches are available to assess the 3D scan accuracy of a test scan compared to a reference. These include linear, angular distances, RMS deviation, and three-dimensional surface deviations, which are other methods to assess scan accuracy^52,54,54^. The current study used RMS values to compare the surface deviations of three-dimensional images, as they have been commonly used to evaluate scan deviations^19,44,46–48,48^, as it is beneficial. In measuring the overall accuracy of the scans^55^.

In this study, the virtual design of the bar was not used as a reference to eliminate the factor of difference in the milling of various materials as a variable. A desktop scan of the milled bar on the same model was used for each group to produce an independent reference^19^. Scanning powder was not employed to exclude the influence of coating thickness^8^. The PEEK bar recommended dimensions from previous literature were a minimum occlusal-cervical height of 5 mm, a minimum anterior buccolingual width of 4 mm, with an increase of width in the areas of the titanium sleeve for a minimum of 6 mm buccolingual width^33,35,36^. The patient’s inter-arch space and clinical situation allowed for a bar substructure design with an occlusal-cervical height of 5.1–6.6 mm and a buccolingual width of 5–6 mm. Buccal and lingual finish lines were created with a width of 1 mm to facilitate measurements and ensure a strong design for the suprastructure material, as shown in Fig. 2. Regarding the titanium bar, smaller dimensions would have been strong enough. However, the exact dimensions were adopted for the titanium bar substructure to standardise the size for both bars. According to reports, the automatic alignment algorithms provide the best possible repeatability^43^. They were therefore used in the present study.

Accurate scanning of the substructure is crucial, as any deviation at this stage is cumulatively transferred to the suprastructure, ultimately impacting its fit and overall prosthetic accuracy.

Airborne particle abrasion was used to improve the optical properties of bars used in this study and to avoid light reflections that the polished surface of the titanium bar might cause^14^.

The titanium framework showed significantly higher trueness and precision than PEEK. This may be attributed to its superior edge clarity, especially when surface-treated using airborne particle abrasion to decrease reflectivity. Revilla-León et al. have suggested that the dark color of the titanium scan body might be the cause^6^. These findings are supported by Baranowski et al.^56^ who stated that the titanium scan body group’s RMS value was lower than that of the PEEK scan body group.

Also, Azevedo et al.^57^, who indicated that the trueness and precision of titanium scanbodies were significantly superior to those of PEEK.

These results contradict those of Lee et al.^12^, and Lorenzo et al.^10^, who stated that PEEK intraoral scan bodies were more accurately scanned than titanium.

The properties of the surface that need to be scanned are a crucial element that may impact point cloud density. It is agreed that the optical properties of the material affect the number of points acquired^13,15,22,23^. In this study, PEEK exhibited better scannability than titanium, probably due to its matte, smooth, and opaque surface. This surface aids light absorption, enabling the scanner to create a denser point cloud. These optical features assist intraoral scanners in gathering additional data points with reduced disruptions, resulting in quicker and more comprehensive surface detection^55,58^. Those results align with Emam et al.^19^, who reported that PEEK was the most scannable material compared to titanium and PMMA. The findings of this investigation demonstrated that PEEK exhibited significantly higher scannability than titanium. Despite PEEK’s benefit in scannability, titanium demonstrated considerably higher accuracy in the final scans. These findings assure the significant difference between scannability concerning the ease and speed of scanning (scannability) and scan accuracy.

The study’s accuracy results are consistent with previously published ranges of whole arch digital implant scans, which show that the precision ranged from 15.2 to 204.2 μm and the trueness of digital implant impressions ranged from 7.6 to 731.7 μm^47,55,58^. To the authors’ knowledge, no study has evaluated the accuracy of intraoral scanning of full arch implant-supported substructures. Accordingly, those ranges were as close as possible.

This research contributes to clinical decision-making, as selecting the most accurately scanned bar material helps achieve highly passive suprastructures. This results in reduced marginal gaps, improved fit, and enhanced long-term serviceability of the final prosthesis. On the other hand, using materials with superior scannability offers significant clinical advantages by reducing chairside time and enabling faster and easier intraoral scanning. However, materials with lower scannability may not be as accurate, which could potentially affect the fit of the definitive restoration. Therefore, when less scannable materials are used, an experienced operator should perform the scanning procedure, utilizing efficient scanning strategies to optimize scan quality and minimize potential errors.

Blender for Dental CAD software program has introduced the I-bar module for designing an inner bar for full arch screw-retained restorations^34^. An inner bar has many benefits, including improving the prosthesis’s strength, providing greater options for suprastructure material selection, reducing weight, and avoiding excessive bulk in either the supra or substructures, improving comfort and esthetics for the patient^34^.

This study agreed with earlier research^7,19,24,43^. that reported that the results of a non-metrology grade software like Medit Link, the 50 μm threshold, are clinically relevant^43^. Further research is required to examine the impact of the difference in the 2 materials’ accuracy and scannability on the accuracy of the produced suprastructure.

This research presents several limitations. Using a single clinical case design inherently restricts the generalisability of the findings. However, this study design was intentionally chosen to facilitate a controlled comparison of the two substructure materials under the same clinical conditions. Intraoral variables, including mucosal colour, soft tissue reflectivity, salivary presence, temperature, humidity, and individual anatomical differences, can affect scan accuracy and the fit of prostheses. Limiting the study to one patient reduced inter-patient variability and allowed for a concentrated examination of the substructure material’s influence. Using only two materials for substructure (bar) fabrication limits the potential outcomes; incorporating additional materials could enhance the results.

## Conclusions

Based on the findings of this study, the following conclusions were drawn:

1. Titanium bar scans showed higher Trueness and precision than PEEK.
2. The PEEK bar substructure was more scannable than titanium and had the least non-captured surface area in mm^2^ in a predetermined time frame.
3. There is no direct correlation between the scanning accuracy of substructure materials and their scannability; materials with high accuracy do not necessarily exhibit superior scannability.

## Supplementary Information

Below is the link to the electronic supplementary material.

Supplementary Material 1

Supplementary Material 2

Supplementary Material 3

Supplementary Material 4

Supplementary Material 5

Supplementary Material 6

## Data Availability

The datasets used and/or analysed during the current study are available. https://docs.google.com/spreadsheets/d/1-XN1x6lElKgRZG_NAMZ1WViRpCR-XAzh/edit?usp=sharing&ouid=108567257312858837033&rtpof=true&sd=true

## Abbreviations

CAD-CAM: Computer-aided design - computer-aided manufacture
PEEK: Poly-ether-ether-ketone
IOS: Intraoral scanner
RMS: Root mean square
PMMA: Poly-methyl- methacrylate
