# Supplementary figures and images for: Influence of substructure material on the scanning accuracy and scannability of implant-supported full arch bar substructures
Source: Sci Rep. 2025 Dec 3;15:43110. doi: 10.1038/s41598-025-28419-2 (PMC12678416; doi:10.1038/s41598-025-28419-2)

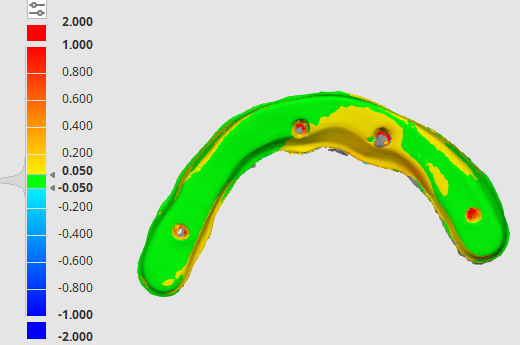

Supplement: Supplementary file 1 — Supplementary Material 1 [file 41598_2025_28419_MOESM1_ESM.png]

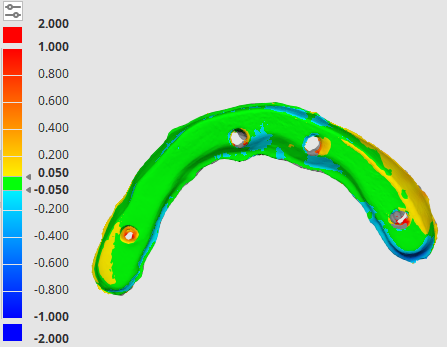

Supplement: Supplementary file 2 — Supplementary Material 2 [file 41598_2025_28419_MOESM2_ESM.png]

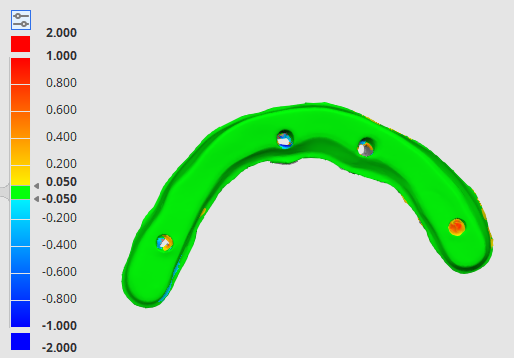

Supplement: Supplementary file 3 — Supplementary Material 3 [file 41598_2025_28419_MOESM3_ESM.png]

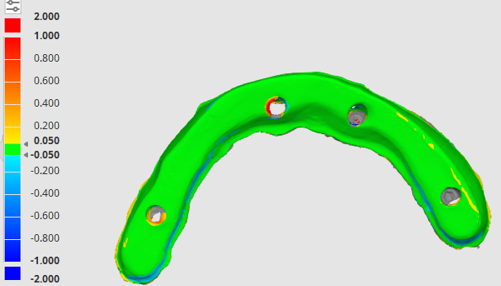

Supplement: Supplementary file 4 — Supplementary Material 4 [file 41598_2025_28419_MOESM4_ESM.png]

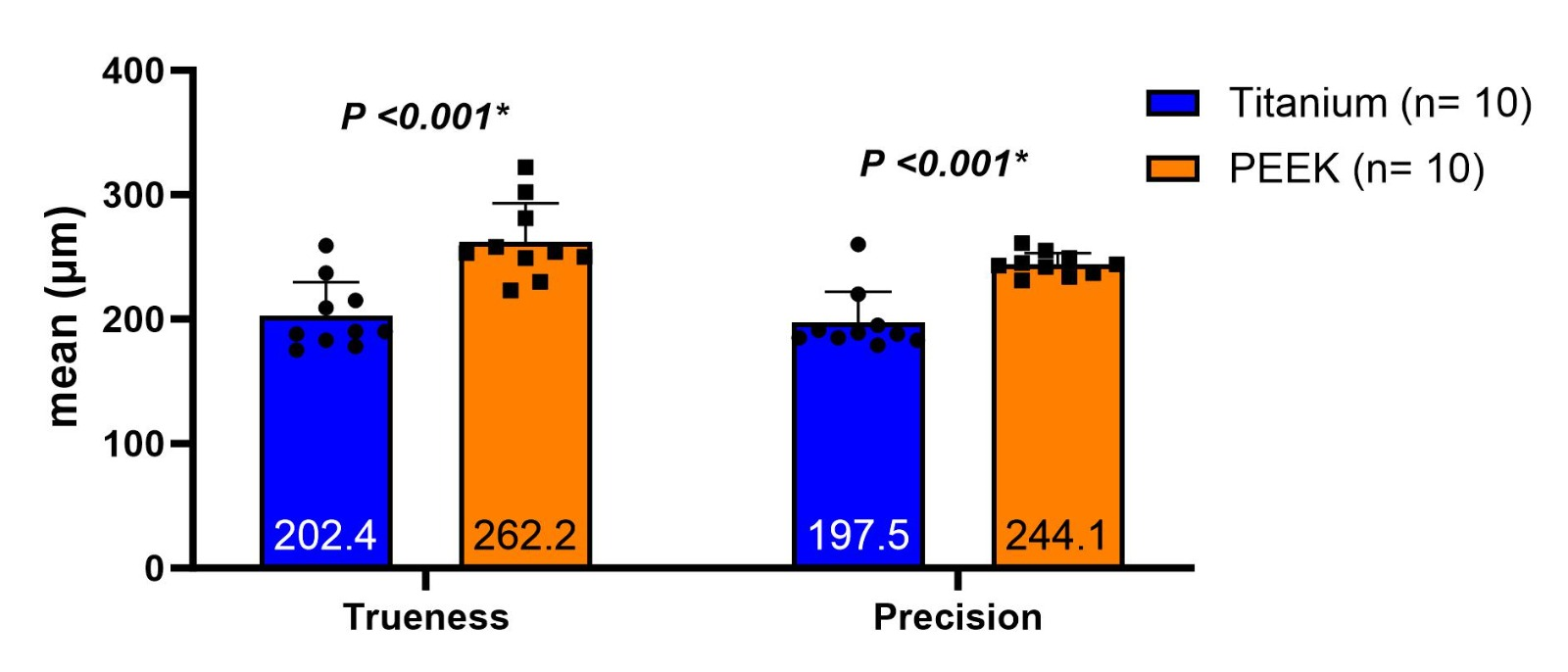

Supplement: Supplementary file 5 — Supplementary Material 5 [file 41598_2025_28419_MOESM5_ESM.png]

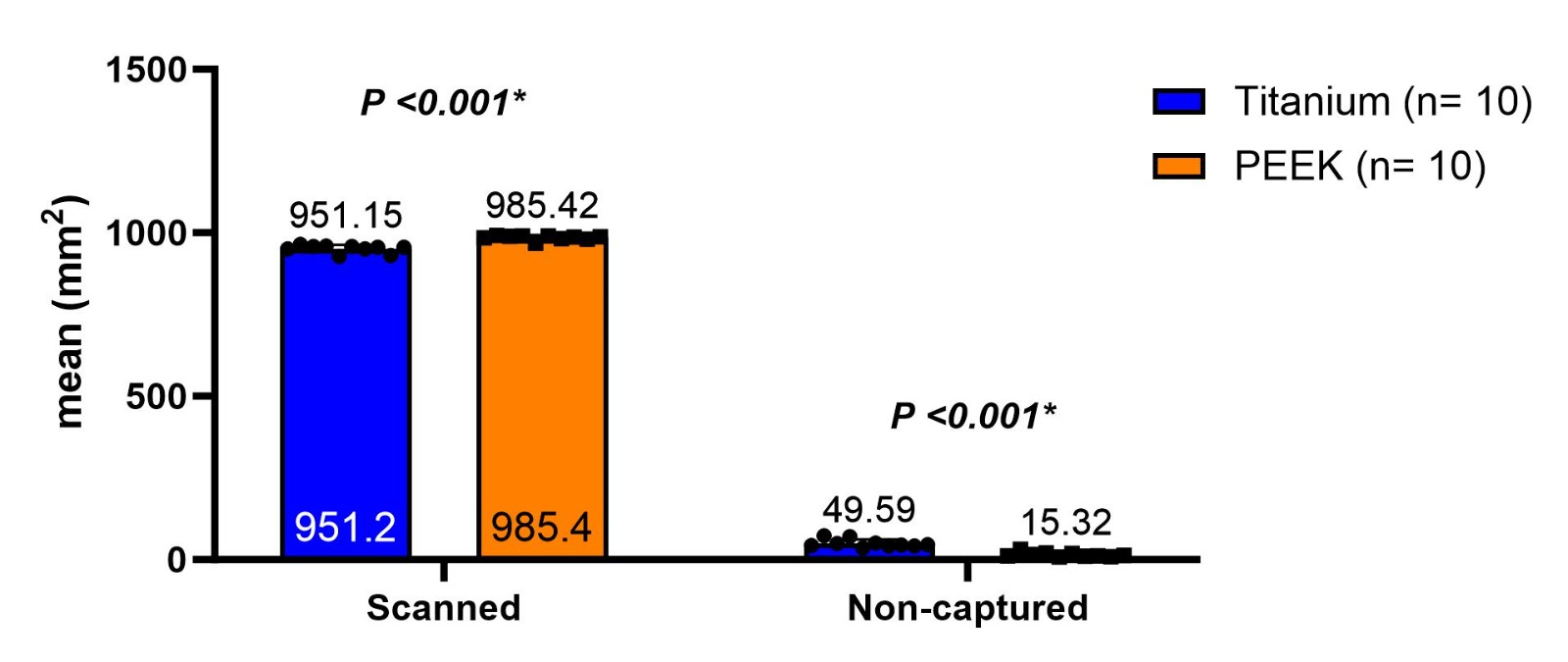

Supplement: Supplementary file 6 — Supplementary Material 6 [file 41598_2025_28419_MOESM6_ESM.png]
